# Pretreatment Neutrophil-to-Lymphocyte Ratio Combined with Platelet-to-Lymphocyte Ratio as a Predictor of Survival Outcomes after Definitive Concurrent Chemoradiotherapy for Cervical Cancer

**DOI:** 10.3390/jcm10102199

**Published:** 2021-05-19

**Authors:** Jeong Won Lee, Ki Ho Seol

**Affiliations:** Department of Radiation Oncology, Daegu Catholic University School of Medicine, Daegu 42472, Korea; gardenlee@cu.ac.kr

**Keywords:** cervical cancer, chemoradiotherapy, neutrophil-to-lymphocyte ratio, platelet-to-lymphocyte ratio

## Abstract

The aim of the study was to evaluate pretreatment neutrophil-to-lymphocyte ratio (NLR) and platelet-to-lymphocyte ratio (PLR) as prognostic factors for predicting clinical outcomes after definitive concurrent chemoradiotherapy (CCRT) for cervical cancer. The cases were divided into two groups based on the values of NLR and PLR: High NLR-PLR (high value in both NLR and PLR) and Low NLR-PLR (low value in either NLR or PLR). The relationships between survival outcomes and the pretreatment NLR-PLR were investigated. Of the 148 patients enrolled in the study, 30 patients died during the median follow-up of 75 months. Based on receiver operating curves, NLR and PLR cut-off values for survival analysis were 2.34 and 148.89. The 10-year overall survival and disease-free survival rates for high NLR-PLR vs. low NLR-PLR were 63.6% vs. 86.2% (*p* = 0.001) and 63.3% vs. 77.5% (*p* = 0.026), respectively. Based on a multivariate analysis, independent predictors of overall survival were high NLR-PLR (hazard ratio [HR], 2.435; 95% confidence interval [CI], 1.106–5.361; *p* = 0.027) and stage (HR 2.659; 95% CI, 1.146–6.613; *p* = 0.024). Increases in both NLR and PLR are associated with poor survival. Elevation in both NLR and PLR before initiation of CCRT may be a useful biomarker for predicting clinical outcomes.

## 1. Introduction

The preferred treatment for patients with locally advanced cervical cancer is definitive radiotherapy with concurrent cisplatin-based chemotherapy [1]. Although definitive concurrent chemoradiotherapy (CCRT) with cisplatin improves the survival of cervical cancer patients, approximately one-third suffer from tumor recurrence or progression [2,3]. The patients with recurrent and metastatic cervical cancer have limited systemic treatment options including combination chemotherapy with the addition of the anti-vascular endothelial growth factor monoclonal antibody, and the prognosis is poor [4,5]. Several clinicopathological factors affect tumor recurrence and survival of cervical cancer patients after treatment, including tumor size, lymph node involvement, and squamous cell carcinoma antigen (SCC-Ag) [6]. Recent investigations have focused on the relationship between tumor recurrence and tumor-associated inflammatory responses [7,8,9]. The inflammatory response plays an important role in the formation and progression of tumors and prognosis [9]. The association of diverse hematologic parameters of systemic inflammation with cancer prognosis has been investigated [8,10]. Among the hematologic parameters, complete blood count with a differential test from routine blood sampling is a simple, cost-effective, and readily available method. Elevated neutrophil-to-lymphocyte ratio (NLR) or platelet-to-lymphocyte ratio (PLR) indicate poor prognosis in various solid tumors [7,11]. Changes in the NLR may be a useful predicting factor in advanced cancer patients treated with anti-PD-1/PD-L1 agents [12]. However, investigations into hematologic parameters for cervical cancer have mainly focused on NLR and its prognostic value [11]. Only a few studies focus on PLR as a predictor in cervical cancer, and the results of these studies are conflicting [13,14,15]. Furthermore, cut-off levels have not been determined.

The aim of the present study was to assess the prognostic significance of NLR and PLR on treatment outcomes and suggest the optimal cut-off levels in patients with locally advanced cervical cancer.

## 2. Materials and Methods

### 2.1. Patients

The inclusion criteria were as follows: (1) newly diagnosed histologically proven squamous cell carcinoma, adenocarcinoma, or adenosquamous carcinoma of the uterine cervix at our institution between 2008 and 2018; (2) clinical and radiologic FIGO (International Federation of Gynecology and Obstetrics) stage IB-IVA with no other evidence of distant metastasis; (3) treatment using a combination of 3-dimensional conformal external beam radiotherapy (3D-CRT) and concurrent weekly cisplatin followed by high-dose-rate brachytherapy; and (4) Eastern Cooperative Oncology Group performance status 0–2. The exclusion criteria were as follows: (1) incomplete treatment; (2) surgical intervention or other treatment before CCRT; (3) a history of cancer in another organ; (4) incomplete clinical information; and (5) clinical signs of infection or other inflammatory conditions, including pneumonia and articular rheumatism, or hematologic disease before CCRT. Finally, 148 patients were included in the study. The following patient data were collected and analyzed: age, primary tumor size, histological subtype, tumor stage, lymph node status, and pretreatment laboratory blood indicators.

### 2.2. Analysis of Inflammatory Markers

Neutrophil, lymphocyte, and platelet counts were obtained from routine blood tests within a day before treatment. NLR was defined as the absolute neutrophil count divided by the absolute lymphocyte count. The PLR was defined from the differential count as the absolute platelet count divided by the absolute lymphocyte count. Patients were divided into two groups based on the NLR and PLR; the high NLR-PLR group had high NLR and PLR and the low NLR-PLR group had either low NLR or low PLR. The relationships between survival outcomes and the pretreatment NLR and PLR were investigated.

### 2.3. Treatment

All patients received a combination of external-beam radiotherapy (EBRT) and concurrent cisplatin-based chemotherapy, followed by high-dose-rate intracavitary brachytherapy (ICBT). During radiotherapy, chemotherapy with weekly cisplatin (40 mg/m^2^ weekly for 6 weeks) was administered. Patients received a median EBRT dose of 45 Gy (range, 45–50.4 Gy) at 1.8 Gy per fraction with whole pelvic radiotherapy (WPRT) or extended-field pelvic radiotherapy (EF-PRT) by 3D-CRT. After WPRT or EF-PRT, the boost irradiation of 9 Gy (median, range, 5.4–18.0 Gy) by 3D-CRT or intensity-modulated radiotherapy was given at 1.8 Gy or 2 Gy per fraction to lymph node (LN) regions that had significant evidence of carcinoma involvement or LN more than 10 mm on MRI findings, involved parametrium, or involved regions of the pelvic sidewall. After adequate tumor regression, high-dose-rate ICBT was performed twice per week using an iridium-192 remote after-loading technique. The standard prescribed dose for each brachytherapy in our institution was 5 Gy to A-point in six fractions, twice weekly. The median prescribed A-point dose was 30 Gy (range, 25–35 Gy). The median total prescribed A-point radiobiological equivalent dose in 2 Gy fractions (α/β = 10) (EBRT + ICBT) was 84.35 Gy (range, 75.50–105.70 Gy; interquartile range, 81.75–90.6 Gy). The median overall irradiation time was 58 days (range, 45–98 days; interquartile range, 53.75–63.0 days).

### 2.4. Response Evaluation and Follow-Up

All patients were subjected to routine post-CCRT surveillance with physical examination, cervicovaginal cytology, laboratory tests (e.g., SCC-Ag), and imaging studies, including abdominopelvic computed tomography (CT), magnetic resonance imaging (MRI), and positron emission tomography (PET)/CT. After completion of CCRT, the patients were evaluated every 3 months for the first 2 years and every 6 months thereafter. Recurrence was diagnosed through physical examination and diagnostic imaging (contrast-enhanced CT, MRI, and/or PET/CT scans) and was confirmed histologically via needle aspiration or excisional biopsy when possible.

### 2.5. Endpoint and Statistical Methods

The primary endpoint was the overall survival (OS) rate. The secondary endpoint was the disease-free survival (DFS) rate. We calculated all occurrences from the date of diagnosis to the date of relapse or the last date of follow-up. Deaths from other causes were censored at the time of the last follow-up.

Intergroup differences in continuous variables were compared using t-tests or Mann–Whitney U tests, as appropriate. Intergroup differences in categorical data were analyzed by chi-square tests, Mann–Whitney U tests, or Fisher’s exact tests, as appropriate. Receiver operated characteristics (ROC) curves were constructed to determine the cut-off values for NLR and PLR that yield the joint maximum sensitivity and specificity. The survival analysis was based on the life-table Kaplan–Meier method. Survival comparisons between groups were made using the log-rank test. A multivariate analysis was performed using the Cox proportional hazard model to predict survival. Two-sided tests were performed and *p*-values < 0.05 were considered statistically significant. Statistical analysis was performed using SPSS ver. 19 (SPCC Inc., Chicago, IL, USA).

## 3. Results

### 3.1. Patient Characteristics and Grouping

The analysis included 148 patients. Patient characteristics are shown in Table 1. Most patients (84.5%) presented with squamous cell carcinoma and 74.3% of patients were stage IIB or a more advanced stage. After completion of CCRT, radiologically complete remission was attained in 145 patients and radiologically partial remission was attained in 3 patients. Of the three patients who showed partial remission, one underwent a pelvic exenteration and the other two underwent adjuvant chemotherapy.

Based on the ROC analysis, NLR and PLR cut-off values for the survival analysis were 2.34 (area under the curve (AUC) 0.643) and 148.89 (AUC 0.632). The patients were divided into a high NLR-PLR group (NLR ≥ 2.34 and PLR ≥ 148.89) with 52 patients and a low NLR-PLR group (NLR < 2.34 or PLR < 148.89) with 96 patients. Patients in the high NLR-PLR group had relatively advanced FIGO stage, low pretreatment hemoglobin, and high pretreatment levels of SCC-Ag compared to these parameters in the low NLR-PLR group (*p* = 0.023, 0.004, and 0.049, respectively). No significant differences in age, histologic distribution, tumor size, or presence of lymph node metastasis were noted between the groups. Characteristics of patients and tumors according to the NLR-PLR groups are shown in Table 2. The prescribed radiation dose and the use of chemotherapeutic agent were not significantly different between the NLR-PLR groups.

### 3.2. Survival Rates and Analysis of Prognostic Factors

The median follow-up was 75 months, and 30 deaths were observed during that time. The 5-year OS and DFS rates for all patients were 81.4% and 78.2%, respectively. Patients with low pretreatment LNR (<2.34) had a significantly better OS (88.9% in 5-year) compared to patients with high pretreatment LNR (74.2% in 5-year) (*p* = 0.017). Patients with low pretreatment PLR (<148.89) had a marginally better OS (87.3% in 5-year) compared to patients with high pretreatment PLR (75.8% in 5-year), but this result was not statistically significant (*p* = 0.051).

**Figure 1:**
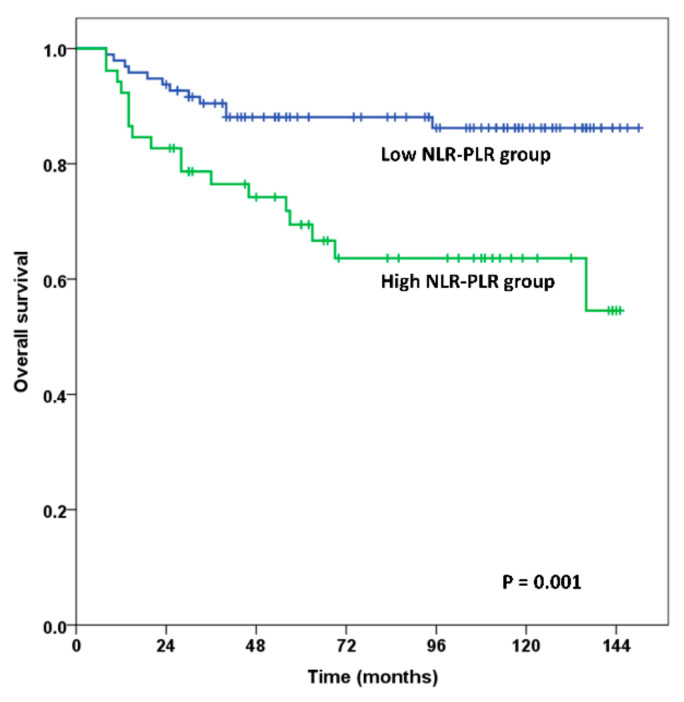
Overall survival estimation using Kaplan–Meier analysis. The 5- and 10-year overall survival rates for the high NLR-PLR group vs. the low NLR-PLR group were 69.4% and 63.6% vs. 88.1% and 86.2% (*p* = 0.001).

**Figure 2:**
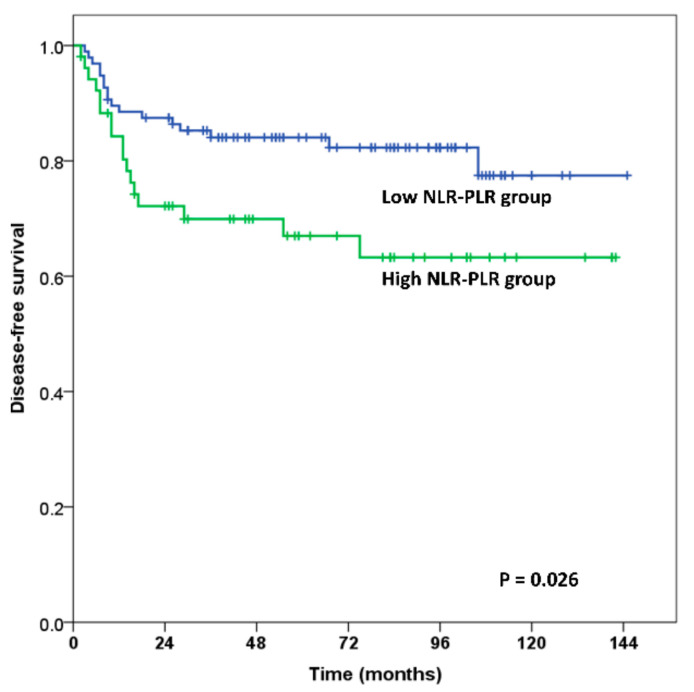
Disease-free survival estimation using Kaplan–Meier analysis. The 5- and 10-year disease-free survival rates for the high NLR-PLR group vs. the low NLR-PLR group were 67.0% and 63.3% vs. and 84.1% and 77.5% (*p* = 0.026).

In the NLR-PLR grouping analysis, the OS and DFS rates were significantly higher in patients with high NLR-PLR compared with the rates in patients with low NLR-PLR. The 5-year and 10-year OS rates were 69.4% and 63.6% in the high NLR-PLR group versus 88.1% and 86.2% in the low NLR-PLR group, respectively (*p* = 0.001 for both) (Figure 1). The 5-year and 10-year DFS rates were 67.0% and 63.3% in the high NLR-PLR group and 84.1% and 77.5% in the low NLR-PLR, respectively (*p* = 0.026 for both) (Figure 2).

Prognostic factors, including age, stage, histologic type, primary tumor size, presence of LN metastasis, standardized uptake value of PET, pretreatment hemoglobin, pretreatment NLR, pretreatment PLR, and pretreatment NLR-PLR group, were analyzed to assess their effects on survival. Prognostic factors related to OS and DFS are shown in Table 3. In the univariate analysis, FIGO stage, presence of LN metastasis, and pretreatment NLR-PLR group were the most significant prognostic factors for OS and DFS. Other factors, including age, primary tumor size, standardized uptake value of PET, and pretreatment SCC-Ag were not statistically significant.

In the multivariate analysis, the independent predictors of OS were high NLR-PLR (hazard ratio [HR], 2.435; 95% confidence interval [CI], 1.106–5.361; *p* = 0.027) and FIGO stage (HR 2.659; 95% CI, 1.146–6.613; *p* = 0.024). The presence of LN metastasis (HR 2.805; 95% CI, 1.359–5.792; *p* = 0.005) was the only independent predictor of DFS, and pretreatment NLR-PLR group was not quite significant (*p* = 0.069). The results are shown in Table 4.

## 4. Discussion

This study revealed that the OS and DFS rate were significantly lower in the high NLR-PLR group compared with the OS and DFS rate in the low NLR-PLR group (*p* = 0.001 for OS, *p* = 0.026 for DFS; Figure 1 and Figure 2). High NLR-PLR was an independent predictor of overall survival (HR, 2.435; 95% CI, 1.106–5.361; *p* = 0.027) in the multivariate analysis.

Systemic inflammation is relevant to the tumor environment, and inflammatory cells, cytokines, and chemokines are involved in creating the tumor environment [9,16]. In particular, neutrophils and platelets promote tumor proliferation and migration, while lymphocytes work against tumor cells [17,18]. Therefore, high NLR and PLR may indicate tumor aggressiveness and host immunity. Several studies have been conducted to evaluate NLR and/or PLR as prognostic factors in cervical cancer [11,13,14,19,20,21,22,23,24,25]. The studies, listed in Table 5 show the connection between prognosis in cervical cancer patients and NLR and/or PLR. Most of these studies analyzed NLR and PLR separately. Only two studies, the Chen et al. study [14] and the present study, combined NLR and PLR in the analysis; these studies verified the efficacy of combined NLR and PLR for predicting survival outcomes in cervical cancer. Chen et al. [14] included patients who underwent radical surgery +/− adjuvant RT or CCRT, whereas the present study targeted patients who received definitive CCRT.

Unlike tumor-specific markers, there are no consensus cut-off values for hematologic parameters. The values ranged from 1.6 to 4 for NLR and 133.02 to 210 for PLR (Table 5). Lee et al. reported reference values of 1.65 for NLR and 132.40 for PLR in healthy South Korean adults [26]. Drugs, cardiovascular, cerebrovascular, liver, and inflammatory diseases, and infection may increase hematologic parameters, including NLR and PLR [27,28,29]. NLR and PLR values may also be affected by the co-existence of these conditions. Therefore, patients with benign inflammatory conditions such as infection, rheumatoid disease, or hematologic disease before treatment were excluded from the study.

Patients were grouped based on pretreatment cut-off NLR and PLR levels of 2.34 and 148.89 (high NLR-PLR group, pretreatment NLR ≥ 2.34 and PLR ≥ 148.89; low NLR-PLR group, pretreatment NLR < 2.34 or PLR < 148.89). These cut-off values are similar to the values reported by Zhang et al. (NLR cut-off value, 2.213; PLR cut-off value, 150.9) [19]. OS was worse for patients in the high NLR-PLR group compared with patients in the low NLR-PLR group, based on univariate and multivariate analyses. Despite the significant difference in the univariate analysis, DFS in the high NLR-PLR group was not significantly different from DFS in the low NLR-PLR group in the multivariate analysis. High NLR-PLR values correlated with advanced FIGO stage, low pretreatment hemoglobin, and a high level of pretreatment SCC-Ag (*p* = 0.023, 0.004, and 0.049, respectively). Thus, cervical cancer patients with advanced FIGO stage, anemia, or elevated SCC-Ag have poor survival outcomes after treatment [30,31,32].

The prognostic importance of the combined NLR and PLR measurements has been demonstrated in a variety of malignant tumors [33,34,35]. In cervical cancer, Onal et al. demonstrated a relationship between pretreatment NLR linked with PLR and treatment outcomes, including tumor burden and tumor response; Onal et al. reported cut-off values for NLR and PLR of 3.11 and 131.18 for predicting disease relapse [13]. Neutrophils and platelets may act together to resist chemotherapy [34,36]. Thus, scoring concepts using NLR and PLR were developed to predict gastric cancer. Consequently, patients with high NLR-PLR values tend to have higher tumor invasiveness [22]. Poor survival outcomes in the high NLR-PLR group in our study may be explained by the above-mentioned reasons.

The usefulness of blood test measurements during and after treatment has been demonstrated [24,37]. However, only a few studies used hematologic parameters obtained before treatment [11,13,14,19,20,21,22,23,24,25]. Zhang et al. investigated preoperative inflammatory hematologic parameters and the pathologic features of the cervical cancer patients treated with primary radical surgery; high NLR and PLR were related to bigger tumor size, deeper invasion of the stroma, and lymph node metastasis [19]. Thus, we infer that inflammatory markers measured before treatment reflect the nature of the intact initial tumor.

There are several limitations to this study due to the retrospective design and relatively small number of patients. Nevertheless, the follow-up duration of 75 months (median) was relatively long compared with other established studies (31.7 to 66 months, Table 5). In addition, we tried to include patients who received identical treatment for cervical cancer.

In conclusion, when the cut-off values of pre-CCRT NLR and PLR were set at 2.34 and 148.89, respectively, the combination of high pre-CCRT NLR and PLR was an independent prognostic factor for survival outcome in locally advanced cervical cancer. An increase in both pre-CCRT NLR and PLR predicts poor DFS and OS in locally advanced cervical cancer patients treated with definitive CCRT. Therefore, this hematologic parameter can be used to identify patients with poor prognoses in need of a more aggressive treatment approach.

## Figures and Tables

**Table 1:** Characteristics of patients and tumors.

Characteristics	Value (%)
Age (years, mean ± SD)	54.2 ± 12.4
Primary tumor size (mm, mean ± SD)	40.1 ± 12.7
Histopathology	
SCC	125 (84.5)
Adenocarcinoma or ASC	23 (15.5)
FIGO Stage	
IB	25 (16.9)
IIA	13 (8.8)
IIB	32 (21.6)
IIIA	0 (0)
IIIB	7 (4.7)
IIIC	67 (45.3)
IVA	4 (2.7)
Lymph node metastasis	
None	78 (52.7)
Pelvic	58 (39.2)
Para-aortic +/− Pelvic	12 (8.1)
SUVmax of PET in primary lesion (median, range)	9.84 (1.88–34.11)
Pretreatment hematologic parameter	
Hemoglobin (g/dL, mean ± SD)	12.0 ± 1.8
SCC Ag. Level (ng/mL, median, range)	4.7 (0.5–69.9)
NLR (median, range)	2.34 (0.99–12.06)
PLR (median, range)	148.89 (58.47–363.45)

Abbreviations: SD, standard deviation; SCC, squamous cell carcinoma; ASC, adenosquamous carcinoma; FIGO, The International Federation of Gynecology and Obstetrics; SUVmax, maximum standardized uptake value; PET, Positron Emission Tomography; SCC Ag., Squamous cell carcinoma related antigen; NLR, neutrophil-lymphocyte ratio; PLR, platelet-lymphocyte ratio.

**Table 2:** Characteristics of patient and tumors according to NLR-PLR groups.

Variables	Low NLR or Low PLR (*n* = 96); *n* (%)	High NLR and High PLR (*n* = 52); *n* (%)	*p* Value
Age (years, mean ± SD)	54.65 ± 12.32	52 ± 12.55	0.532
Histopathology			
Squamous cell carcinoma	83 (66.4)	42 (33.6)	0.362
Adenocarcinoma or ASC	13 (56.5)	10 (43.5)	
FIGO stage			
IB-IIB	52 (74.3)	18 (25.7)	0.023
III-IVA	44 (56.4)	34 (43.6)	
Tumor size, cm			
<4	51 (71.8)	20 (28.2)	0.088
≥4	45 (58.4)	32 (41.6)	
Lymph node metastasis			
Absent	53 (67.9)	25 (32.1)	0.407
Present	43 (61.4)	27 (38.6)	
hemoglobin (g/dL, mean ± SD)	12.27 ± 1.15	11.39 ± 2.22	0.004
SCC Ag. Level (ng/mL, median, range)	4.1 (0.5–48.2)	8.6 (0.3–69.9)	0.049

Abbreviations: NLR, neutrophil-lymphocyte ratio; PLR, platelet-lymphocyte ratio; SD, standard deviation; ASC, adenosquamous carcinoma; FIGO, The International Federation of Gynecology and Obstetrics; SCC Ag., Squamous cell carcinoma related antigen.

**Table 3:** Univariate survival analysis.

Variable	Number of Patients	OS (%)		*p* Value	DFS (%)		*p* Value
		5-Year	10-Year		5-Year	10-Year	
Age (years)							
<60	100	84.7	83.1	0.082	80.6	73.0	0.620
≥60	48	74.4	68.4		73.2	73.2	
FIGO Stage							
IB and II	70	90.9	89.0	0.002	88.1	80.0	0.007
III and IVA	78	72.8	67.5		69.1	65.6	
Pathologic type							
SCC	125	81.9	77.9	0.787	81.5	74.9	0.024
AC/ASC	23	78.3	78.3		60.6	60.6	
Tumor size							
<4 cm	71	84.3	78.0	0.933	79.4	71.7	0.775
≥4 cm	77	78.6	78.6		76.9	74.4	
Lymph node metastasis							
Absent	78	87.8	86.2	0.009	88.0	81.4	0.002
Present	70	74.1	67.5		66.9	62.4	
Pretreatment Hb.							
Normal	84	87.9	87.9	0.006	77.8	68.8	0.902
Low (<12 g/dL)	64	72.9	66.1		78.9	76.0	
Pretreatment NLR							
<2.34	74	88.9	86.3	0.017	80.9	74.1	0.415
≥2.34	74	74.2	70.2		75.3	70.6	
Pretreatment PLR							
<148.89	74	87.3	84.7	0.051	83.6	72.2	0.243
≥148.89	74	75.8	71.9		72.7	70.3	
Pretreatment NRL-PLR group							
Low NLR or PLR	96	88.1	86.2	0.001	84.1	77.5	0.026
High NLR and PLR	52	69.4	63.6		67.0	63.3	

Abbreviations: OS, overall survival; DFS, disease free survival; FIGO, The International Federation of Gynecology and Obstetrics; SCC, squamous cell carcinoma; AC, adenocarcinoma; ASC, adenosquamous carcinoma; SUVmax, maximum standardized uptake value; PET, Positron Emission Tomography; SCC Ag., Squamous cell carcinoma related antigen; Hb, hemoglobin; NLR, neutro-phil-lymphocyte ratio; PLR, platelet-lymphocyte ratio; SD, standard deviationOS, overall survival; DFS, disease free survival; FIGO, The International Federation of Gynecology and Obstetrics; SCC, squamous cell carcinoma; AC, adenocarcinoma; ASC, adenosquamous carcinoma; LN, lymph node; SUVmax, maximum standardized uptake value; PET, Positron Emission Tomography; SCC Ag., Squamous cell carcinoma related antigen; Hb, hemoglobin; NLR, neutrophil-lymphocyte ratio; PLR, platelet-lymphocyte ratio; SD, standard deviation.

**Table 4:** Multivariate survival analysis.

Variables	Risk Factors	HR (95% CI)	*p* Value
Overall Survival			
FIGO Stage	IB, II vs. III, IVA	2.752 (1.146–6.613)	0.024
Pretreatment NLR-PLR group	low vs. both high group	2.435 (1.106–5.361)	0.027
Disease-free survival			
Lymph node metastasis	Absent vs. Present	2.805 (1.359–5.792)	0.005
Pretreatment NLR-PLR group	low vs. both high group	1.884 (0.952–3.727)	0.069

Abbreviations: HR, hazard ratio; CI, confidence interval; FIGO, The International Federation of Gynecology and Obstetrics; NLR, neutrophil-lymphocyte ratio; PLR, platelet-lymphocyte ratio; SCC, squamous cell carcinoma; AC, adenocarcinoma; ASC, adenosquamous carcinoma.

**Table 5:** Multivariate Studies analyzing the association between the prognosis and pretreatment NLR and/or PLR in cervical cancer ^1^.

Study	Patient Number	Histologic Type	FIGO Stage	Main Treatment Modality	Prognostic Parameter	NLT Cut-Off Value	PLR Cut-Off Value	Follow-Up Duration (Median, Months)	Results (Significant Parameter)	
									Univariate Analysis	Multivariate Analysis
Zhang et al. [19]	460	SCC, AC	I-II	OP ± RT	NLR, PLR	2.213	150.9	69	NLR: DFS, OS	NLR: DFS
Onal et al. [13]	235	SCC, AC	IB2-IVA	CCRT + BT	NLR, PLR	3.03	3.03	31.7	NLR: DFS, OS PLR: OS	NLR: DFS, OS
Chen et al. [14]	407	SCC, non-SCC	IB-IIA	OP ± RT/CCRT	NLR, PLR, combined NLR and PLR	2.59 for DFS, 2.09 for OS	152.02 for DFS 2.09 for OS	NR	NLR, PLR, Combined NLR and PLR ^2^: DFS, OS	
Jonska-Gmyrek et al. [20]	94	AC	IA-IV	Stage IA, IB1, IIA: OP + RT/CCRT. Stage IB2, IIB-IV: RT/CCRT	NLR, PLR	1.6	158	66	NLR: DFS PLR: DFS for patients with OP + RT/CCRT	NLR: DFS, OS for all patients PLR: OS for stage IIB-IV
Holub et al. [21]	151	SCC, AC, OTC	I-IV	RT and/or CTx, and/or OP	NLR, PLR	3.8	210.0	43.8	NLR, PLR: OS	(not significant)
Prabawa et al. [22]	282	SCC, AC	I-IV	NR	NLR, PLR	3.38	172.05	NR	NLR, PLR: associated with cervical cancer invasiveness	
Trinh et al. [24]	99	SCC, AC, ASC, CIN III, OTC	I-IV	CCRT + BT	NLR, PLR	1.65	186.93	48.99 ^3^	NLR: DFS, OS	
Lima et al. [25]	102	SCC, AC	I-IV	Stage I: OP Stage IIA: OP + (RT and/or CTx) ^4^. Stage IIB-IV: CCRT.	NLR, PLR	4	165.45	NR	NLR, PLR: DFS, OS	NLR: DFS, OS
Current study	148	SCC, AC, ASC	IB-IVA	CCRT + BT	Combined NLR and PLR	2.34	148.89	75	DFS, OS	OS

^1^ The parameters most important to this Table were pretreatment NLR and PLR among the several hematologic parameters. ^2^ Combined NLR and PLR were more significantly associated with predicting DFS and OS. ^3^ Converting years to months. ^4^ They commented that 40 (39.2 %) received surgery and 62 (60.8 %) patients received radiotherapy and/or chemotherapy among all patients. Abbreviations: NLR, neutrophil-to-lymphocyte ratio; PLR, platelet-to-lymphocyte ratio; FIGO, International Federation of Gynecology and Obstetrics; SCC, squamous cell carcinoma; AC, adenocarcinoma; OP, operation (=radical surgery); RT, radiotherapy; DFS, disease free survival; OS, overall survival; CCRT, concurrent chemoradiotherapy; BT, brachytherapy; NR, not reported; OTC, other type of carcinoma; CTx, chemotherapy; ASC, adenosquamous carcinoma; CIN III, cervical intraepithelial neoplasia grade 3.

## Data Availability

The data that support the findings of this study are available on request from the corresponding author. The data are not publicly available due to privacy or ethical restrictions.
